# Reduced Brain Cortex Angiogenesis in the Offspring of the Preeclampsia-Like Syndrome

**DOI:** 10.1161/HYPERTENSIONAHA.123.21756

**Published:** 2023-09-28

**Authors:** Felipe Troncoso, Hermes Sandoval, Belén Ibañez, Daniela López-Espíndola, Francisca Bustos, Juan Carlos Tapia, Pedro Sandaña, Esthefanny Escudero-Guevara, Francisco Nualart, Eder Ramírez, Robert Powers, Manu Vatish, Hiten D. Mistry, Lesia O. Kurlak, Jesenia Acurio, Carlos Escudero

**Affiliations:** Vascular Physiology Laboratory, Department of Basic Sciences, Universidad del Bío-Bío, Chillán, Chile; Escuela de Tecnología Médica, Facultad de Medicina, Universidad de Valparaíso, Chile; Group of Research and Innovation in Vascular Health, Chillan, Chile; Escuela de Tecnología Médica, Facultad de Medicina, Universidad de Valparaíso, Chile; Stem Cells and Neuroscience Center, School of Medicine, University of Talca, Chile; Anatomopatholy Unit, Hospital Clinico Herminda Martin, Chillan, Chile; Vascular Physiology Laboratory, Department of Basic Sciences, Universidad del Bío-Bío, Chillán, Chile; Laboratory of Neurobiology and Stem Cells NeuroCellT, Department of Cellular Biology, Center for Advanced Microscopy CMA Bio-Bio, Faculty of Biological Sciences, University of Concepcion, Chile; Departamento de Biología Celular, Facultad de Ciencias Biológicas, Universidad de Concepción, Chile; Laboratory of Neurobiology and Stem Cells NeuroCellT, Department of Cellular Biology, Center for Advanced Microscopy CMA Bio-Bio, Faculty of Biological Sciences, University of Concepcion, Chile; Magee-Womens Research Institute, Department of Obstetrics, Gynecology and Reproductive Sciences, https://ror.org/01an3r30 University of Pittsburgh, PA; Nuffield Department of Women’s Health and Reproductive Research, University of Oxford, England; Division of Women and Children’s Health, School of Life Course and Population Sciences, King’s College London, United Kingdom; Stroke Trials Unit, School of Medicine, University of Nottingham, United Kingdom; Vascular Physiology Laboratory, Department of Basic Sciences, Universidad del Bío-Bío, Chillán, Chile; Vascular Physiology Laboratory, Department of Basic Sciences, Universidad del Bío-Bío, Chillán, Chile; Group of Research and Innovation in Vascular Health, Chillan, Chile

**Keywords:** actins, brain fetal development, cell movement, humans, preeclampsia, pregnancy

## Abstract

**Background:**

Children from pregnancies affected by preeclampsia have an increased risk of cognitive and behavioral alterations via unknown pathophysiology. We tested the hypothesis that preeclampsia generated reduced brain cortex angiogenesis in the offspring.

**Methods:**

The preeclampsia-like syndrome (PELS) mouse model was generated by administering the nitric oxide inhibitor NG-nitroarginine methyl ester hydrochloride. Confirmatory experiments were done using 2 additional PELS models. While in vitro analysis used mice and human brain endothelial cells exposed to serum of postnatal day 5 pups or umbilical plasma from preeclamptic pregnancies, respectively.

**Results:**

We report significant reduction in the area occupied by blood vessels in the motor and somatosensory brain cortex of offspring (postnatal day 5) from PELS compared with uncomplicated control offspring. These data were confirmed using 2 additional PELS models. Furthermore, circulating levels of critical proangiogenic factors, VEGF (vascular endothelial growth factor), and PlGF (placental growth factor) were lower in postnatal day 5 PELS. Also we found lower VEGF receptor 2 (KDR [kinase insert domain-containing receptor]) levels in mice and human endothelial cells exposed to the serum of postnatal day 5 PELS or fetal plasma of preeclamptic pregnancies, respectively. These changes were associated with lower in vitro angiogenic capacity, diminished cell migration, larger F-actin filaments, lower number of filopodia, and lower protein levels of F-actin polymerization regulators in brain endothelial cells exposed to serum or fetal plasma of offspring from preeclampsia.

**Conclusions:**

Offspring from preeclampsia exhibited diminished brain cortex angiogenesis, associated with lower circulating VEGF/PlGF/KDR protein levels, impaired brain endothelial migration, and dysfunctional assembly of F-actin filaments. These alterations may predispose to structural and functional alterations in long-term brain development.

Preeclampsia is a life-threatening pregnancy syndrome characterized by new onset hypertension after 20 weeks of gestation accompanied by proteinuria or evidence of maternal organ injury^1,2^ and increases the risk of stroke as adults.^3^ Moreover, preeclampsia doubles the risk of neurocognitive and mood disorders in children.^4–6^ However, the underlying mechanisms of these last associations, despite being investigated in animal studies,^7–10^ are largely unknown.

Brain vascular beds ensure brain homeostasis. Hence, alterations in cerebrovascular formation or function occurring during development may have lasting consequences for neurodevelopment. To our knowledge, whether brain blood vessel formation (ie, brain angiogenesis) is impaired in offspring exposed to preeclampsia is unknown. Despite some indirect pieces of evidence have correlated the cognitive performance of children born to women with preeclampsia with reduction in cerebral vessel radii in the occipital and parietal lobes.^11^ Animal studies have also shown defects in the structure and function of the middle cerebral artery in offspring from rat preeclampsia-like syndrome (PELS) model.^12,13^ More recently, we found that mice (postnatal day 5 [P5]) of PELS (characterized by a reduction in placental perfusion) showed a significant decrease in brain perfusion, which may involve structural alterations in the brain microcirculation.^10^

Vascular endothelial growth factor (VEGF), placental growth factor (PlGF), and VEGF receptors (VEGFRs) are critical regulators of angiogenesis.^14^ These proteins and sFLT1 (soluble fms-like tyrosine kinase; a decoy receptor of VEGF) play critical roles in angiogenesis and neurogenesis.^15,16^ Elevated levels of sFLT1 in blood collected from the umbilical cord,^17,18^ newborns,^19^ or in adults (between 20 and 30 years old), as well as lower circulating levels of VEGF,^19^ are reported in children born from women with preeclampsia.^20^ These data suggest that an imbalance between proangiogenic and antiangiogenic factors may impair the function of fetal endothelial cells. However, whether this imbalance impairs brain angiogenesis is unclear.^21^ Although genetic deletion of PlGF leads to impaired brain vascular development, including unilateral hypoplasia and less collateral vessel formation (ie, angiogenesis) in the circle of Willis, which initiates at the embryonic stage and persists after birth.^14^ At the same time, deletion of VEGF is lethal due to defects in the vasculature.^22^ In addition, less attention has been given to the VEGFR2 or the KDR (kinase insert domain-containing receptor) in preeclampsia. These receptors are also critically involved in endothelial, neurovascular, and neuronal functions.^15,16^ However, there are no studies regarding the potential role of KDR in brain angiogenesis in offspring from preeclampsia.

Importantly, VEGF is a critical factor in endothelial sensing of promigratory signals via the formation of cell filopodia in the brain cortex.^23^ In addition, migration of endothelial cells early after birth (up to postnatal day 7) is critical for adequate brain angiogenesis processes in the neonatal stage in mice.^24^ Both cell migration and filopodia formation require adaptation of the cytoskeleton, which occurs via dynamic changes in the F-actin polymerization. Regulators of F-actin polymerization include the complex of ARP2/3 (actin-related proteins 2 and 3), cofilin (active form), and phosphorylated cofilin (inactive form), which are critical regulators of the disassembly of F-actin filaments.^25^ To date, no information regarding impairment of F-actin polymerization and brain endothelial cell migration has been described in preeclampsia.

Therefore, we investigated whether offspring born from mothers with PELS exhibit impaired brain cortex angiogenesis and dysfunction in endothelial cell migration mediated by the VEGF/VEGFRs signaling pathway and alterations in the disassembly of F-actin filaments. Such alterations may impact the cognitive abilities of the offspring.

## Methods

## Results

### Preeclampsia-Like Mouse Model

**Figure 1:**
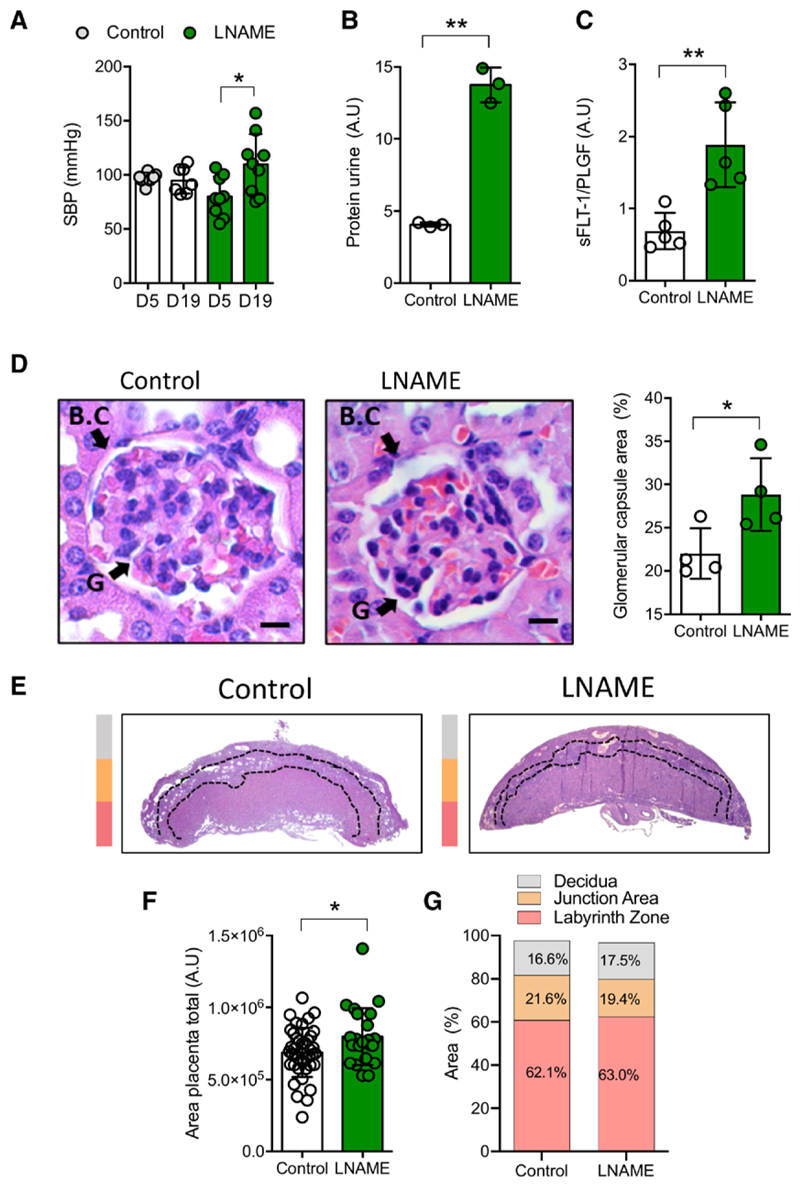
Characterization of the preeclampsia-like syndrome (PELS) using NG-nitroarginine methyl ester hydrochloride (L-NAME) administration in pregnant mice. PELS model was generated by administration of L-NAME (150 μg·kg^−1^·d^−1^) between D7 and D19 of gestation in pregnant mice. **A**, Systolic blood pressure (SBP) at D19 compared with D5 of gestation. **B**, Qualitative analysis of proteinuria. **C**, sFLT1 (soluble fms-like tyrosine kinase)/placental growth factor (PlGF) ratio. **D**, Representative images of hematoxylin/eosin-stained kidneys in control and L-NAME supplemented dams showing the percentage of area occupied by the glomerular capsule area (arrows). **E**, Representative image of hematoxylin/eosin-stained placentas from L-NAME and control pregnancies. Color scale represents respective functional placental structures, such as decidua (gray), junction area (orange), and labyrinth (pink). **F**, Placental area and respective representation of the analyzed placental functional areas. Each dot represents 1 study subject. Values are presented in the median±interquartile range. *P<0.05, **P<0.005.

Compared with control dams, receiving tap water (ie, diluent), NG-nitroarginine methyl ester hydrochloride (L-NAME) administration significantly increases weight gain at day 19 of gestation (Figure S2A). Moreover, L-NAME also increased systolic blood pressure (Figure 1A; n=9; *P*=0.02) and mean arterial blood pressure (Figure S2B; n=9; *P*=0.07) at day 19 compared with day 5 of gestation. PELS dams showed elevated protein levels in the urine (Figure 1B), which were associated with higher sFLT1/PlGF ratio (Figure 1C; Figure S2C and S2D) and structural alterations in the glomerulus, as reduced glomerular area and increased glomerular capsule area (Figure 1D). Also, compatible with clinical findings in humans,^40^ we found higher circulating levels of sFLT1 in the PELS (n=6) than in control dams (n=5; 126.1±72.8 versus 65.3±21.8 pg/mL; *P*=0.02, respectively).

**Figure 2:**
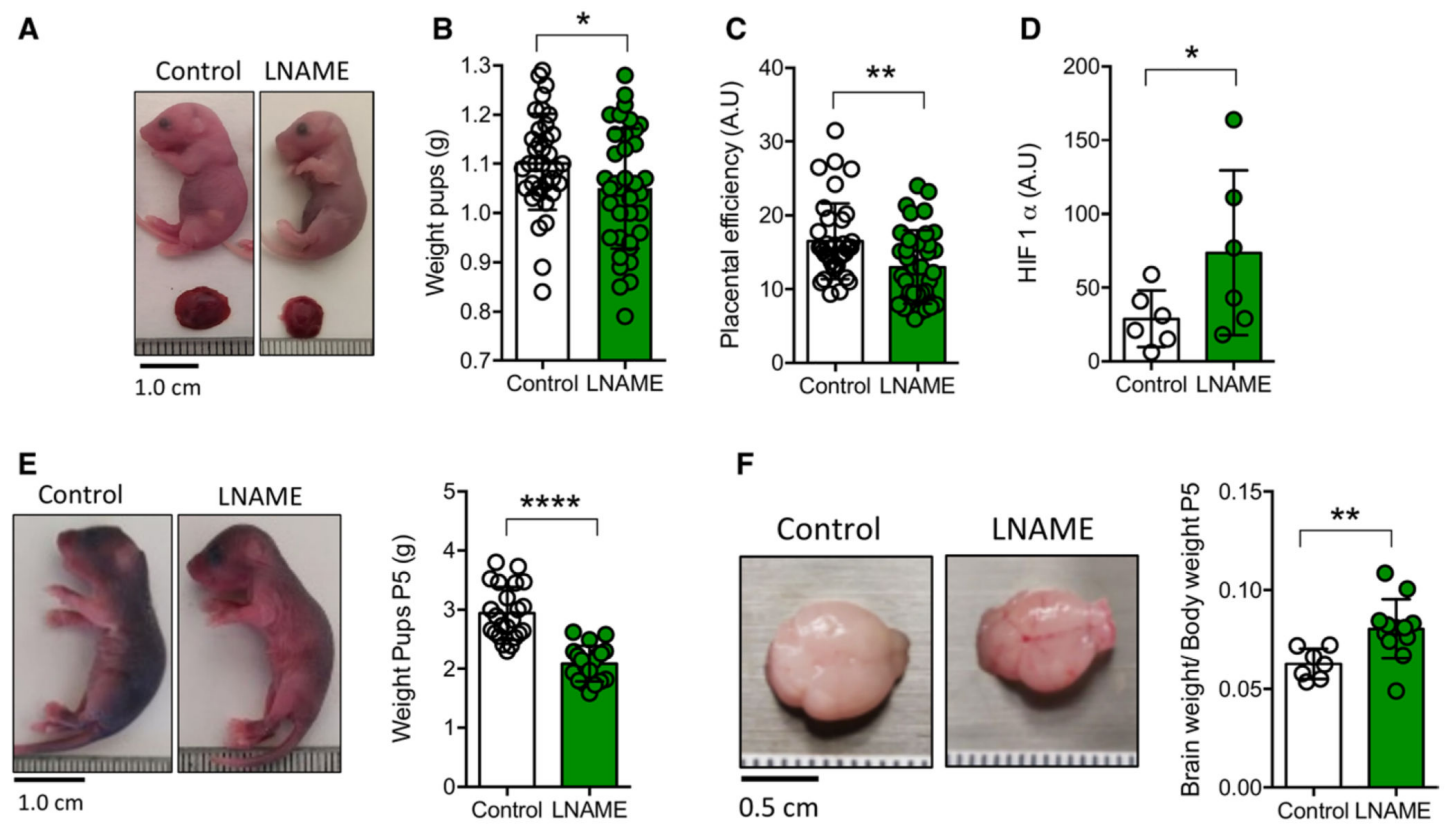
Offspring characteristics in the preeclampsia-like syndrome (PELS) model. **A**, Fetus (day 19 [D19]) from control and NG-nitroarginine methyl ester hydrochloride (L-NAME) dams. **B**, Weight of D19 fetuses. **C**, Placental efficiency (estimated for the placental weight/fetal weight ratio). **D**, Relative values of HIF-1α (hypoxia-inducible factor type 1 alpha) protein amount in placentas analyzed by immunohistochemistry. **E**, Pups (postnatal day 5 [P5]) from control and L-NAME dams, showing reduced weight in the latter. **F**, Representative images of pup’s brains (P5), showing high brain weight/body weight ratio in offspring from L-NAME dams. Each dot represents 1 study subject. Values are presented in the median±interquartile range. *P<0.05, **P<0.005, ****P<0.0001.

No significant differences were found in the total number of pups or male or female pups per litter between groups (Figure S2E through S2G). Placental weight was higher in the PELS group than in controls (90±30 versus 72±21 mg; *P*=0.003; n=37 per group). Accordingly, placental morphology analysis showed a greater placenta area without significant differences in the distribution of the decidual, junctional, or labyrinth areas (Figure 1E and 1F). Nevertheless, PELS fetuses (at day 19) were smaller than controls (Figure 2A and 2B). Therefore, these placenta/fetus weight mismatch significantly reduced placental efficiency in the PELS group (Figure 2C), particularly in male offspring (Figure S2H and S2I). Notably, immunohistochemistry analysis of placentae from the PELS showed significantly high levels of HIF-1α (hypoxia-inducible factor type 1 alpha; Figure 2D). When we analyzed neonatal pups (P5), the offspring from PELS dams (P5-PELS) still had low weights (Figure 2E; Figure S3A and S3B) and shorter lengths in both males and females, compared with their respective controls (Figure S3C through S3E). However, a higher brain/body weight ratio (*P*<0.05) was found in P5-PELS compared with the control group (Figure 2F).

### Analysis of Brain Cortex Angiogenesis

**Figure 3:**
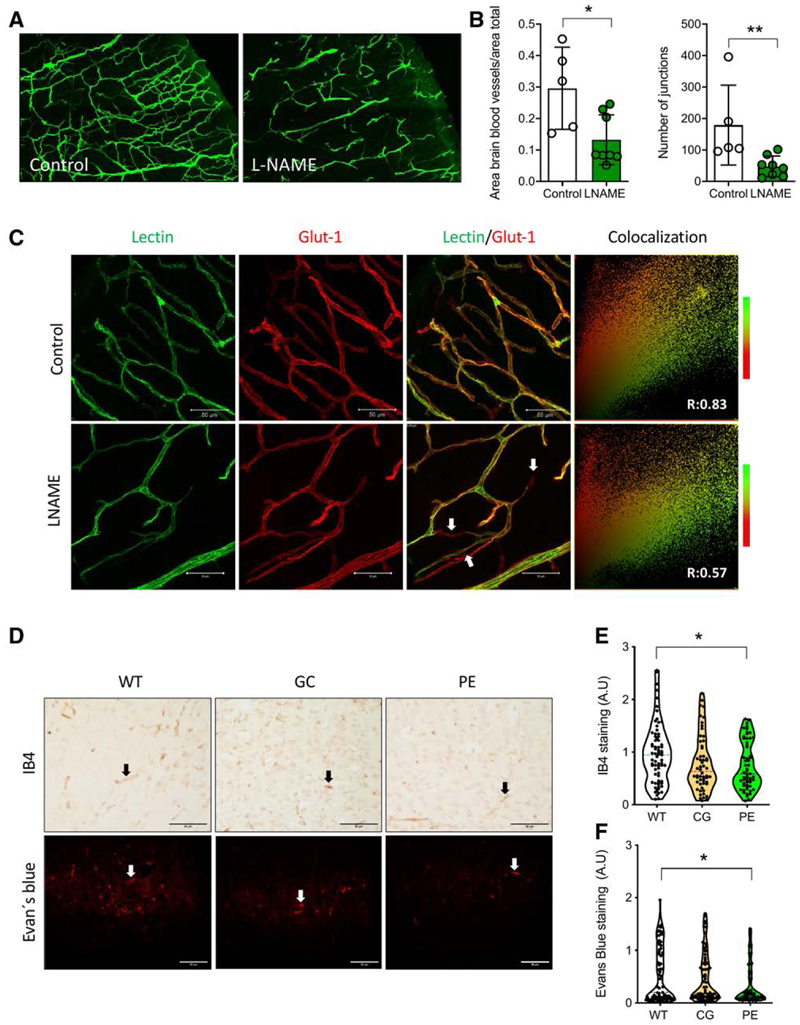
Analysis of brain blood vessels in postnatal day 5 (P5) preeclampsia-like syndrome (PELS). **A**, Representative confocal fluorescence image of motor cortex blood vessels in pups (P5) from control and NG-nitroarginine methyl ester hydrochloride (L-NAME) dams. **B**, Area covered by blood vessels per analyzed brain area and number of blood vessel junctions in P5 from the L-NAME group. **C**, Representative confocal analysis of brain blood vessels with a functional lumen (ie, positive for green, fluorescent lectin, or vascularized) and total blood vessel estimation using GLUT1 (glucose transporter type 1) immunostaining (red). Images of colocalization analysis and Pearson correlation (R) are included. Blood vessels without a functional lumen are indicated with arrows. **D, Top**, Immunostaining for brain blood vessels using IB4 (total blood vessels) or button images with a functional lumen (positive for Evans blue fluorescence) in brain cortex areas (ie, motor and somatosensory areas) of P5 brains of wild type (WT), genetic control (GC), and genetic preeclampsia-like syndrome (PE) mouse models. Analysis of isolectin-IB4 staining (**E**) and Evans blue fluorescence (**F**) per area in P5 of WT, GC, and PE dams. Each dot represents 1 study subject. Values are presented in the median±interquartile range. *P<0.05, **P<0.005. In **E**, the line in each representative image represents 50 μm.

We found that the area occupied by blood vessels in the motor and somatosensory cortex was significantly smaller in P5-PELS offspring compared with the control group (Figure 3A and 3B). We also observed fewer junctions between the brain blood vessels in P5-PELS (Figure 3B). Using double staining with GLUT1 (glucose transporter type 1)—a well-described brain endothelial cell marker^34^—we estimated the total number of blood vessels (ie, GLUT1 positive) and the vessels with a functional lumen (ie, positive to lectin or vascularized vessels) in the brain cortex of P5-PELS. We found a lower total number of blood vessels (ie, GLUT1 positive; Figure 3C; Figure S4A) in the brain cortex of P5-PELS and a reduction in the vascularized vessels (Figure 3C, white arrows). Indeed, the positive correlation between GLUT1 and lectin was lost in the P5-PELS. Reduced GLUT1 in motor and somatosensory P5-PELS brain cortex was further confirmed using Western blotting (Figure S4B and S4C).

To confirm our findings in the P5-PELS L-NAME model, we used 2 additional models of preeclampsia. We, therefore, examined brains from P5 pups from the reduction of uterine perfusion (RUPP) model (Figure S5)^10^ and a genetic model characterized by crossbreeding between male C1q^−/−^ and wild-type females (Figure S6)^39^ and their respective controls. Characterization of RUPP pups was described in our previous publication.^10^ In contrast, morphometric and obstetric characteristics of the pups from the genetic model are included in this article (Figure S6B through S6F) and in a previous publication from our group.^39^ Isolectin B4 (IB4) positive staining was used in those pups’ brains to estimate the total number of blood vessels like GLUT1 reported above. Additionally, we used Evans blue (fluorescent dye), allowing us to detect vascularized vessels.^33^ Pups (P5) from the genetic model of preeclampsia exhibited a significant reduction in the IB4 staining (ie, vascular density) in the motor and somatosensory brain cortex compared with wild-type pups (Figure 3D, top, black arrows; Figure 3E), which was linked to reduced vascularized vessels (Figure 3D, bottom, white arrows; Figure 3F). Consistently, P5 pups from the RUPP model also showed reduced brain vascular density in the motor and somatosensory cortex (IB4 positive area, Figure S5A and S5B) but without changes in the vascularized vessels (Figure S5C).

### VEGF/KDR Imbalance in Offspring From Preeclampsia

**Figure 4:**
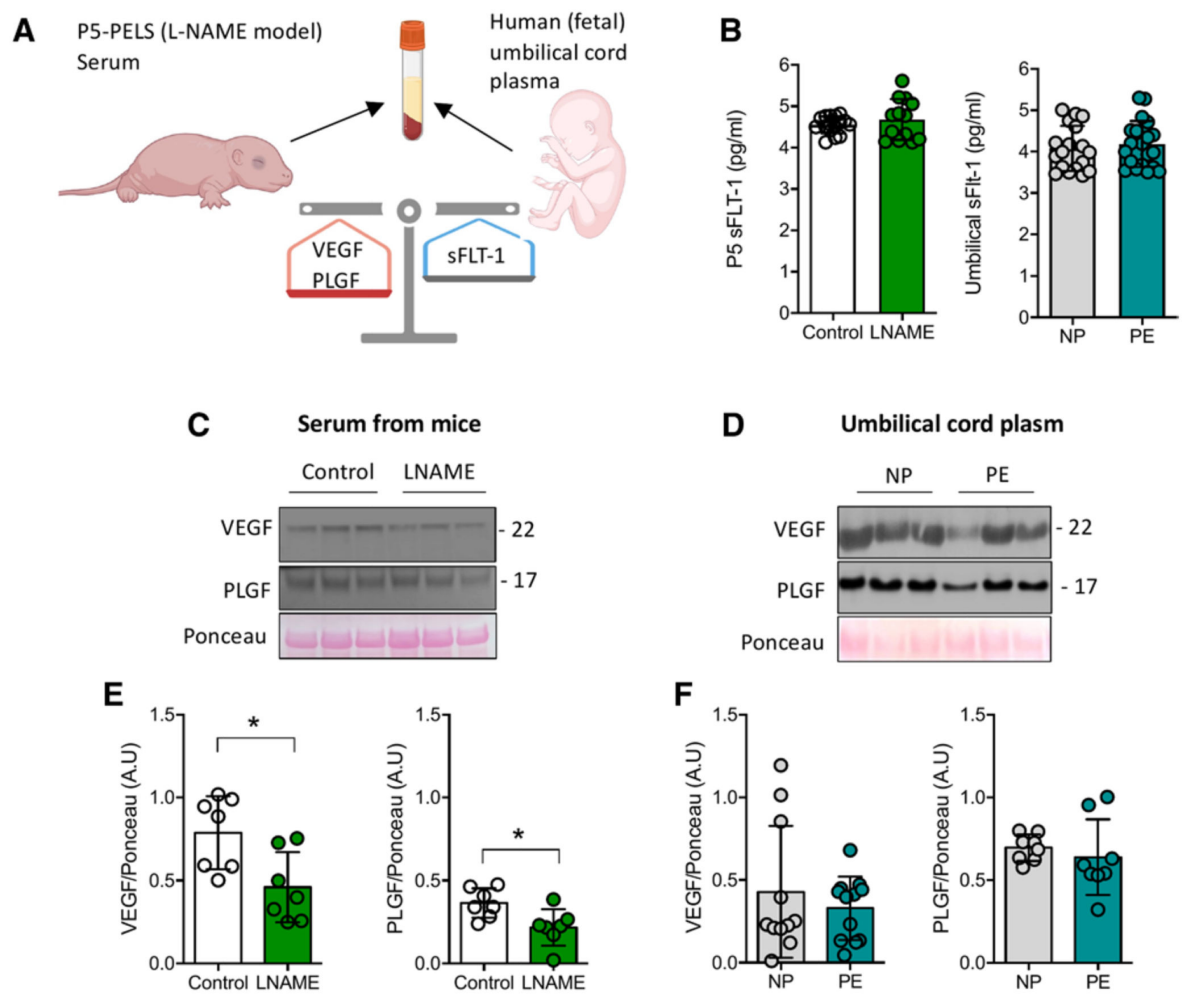
Imbalance of circulating proangiogenic and antiangiogenic markers in offspring from preeclampsia. **A**, Cartoon showing serum or plasma extraction from postnatal day 5 (P5) pups and human umbilical cord (fetal). **B**, sFLT1 (soluble fms-like tyrosine kinase) circulating levels (ie, antiangiogenic protein) in P5 pups from NG-nitroarginine methyl ester hydrochloride (L-NAME) dams or in human umbilical vein plasma (fetal) from normal (NP) or preeclamptic (PE) pregnancies. **C**, Representative images of circulating VEGF (vascular endothelial growth factor) and PLGF (ie, proangiogenic proteins) in serum from P5 pups from control and L-NAME dams or (**D**) fetal plasma from NP and PE. Densitometric analysis of (**E**) VEGF and PLGF in P5 preeclampsia-like syndrome (PELS) and respective controls. **F**, Densitometry of VEGF (n=12 per group; *P*=0.15) and PlGF (placental growth factor; n=8 per group; *P*=0.09) circulating levels. Each dot represents 1 study subject. Values are presented in the median±interquartile range. *P<0.05.

We analyzed the expression of critical regulators of angiogenesis, VEGF, PlGF, and VEGFRs in the serum of offspring from PELS dams (P5-PELS) and their controls, as well as in human umbilical vein plasma (fetal plasma) from normal pregnancy and preeclampsia (Figure 4A; Table S2 for clinical characteristics). Compared with respective controls, no significant changes were found in the circulating levels of sFLT1 (*P*>0.05; Figure 4B) either in serum from P5-PELS or in human fetal plasma from infants born from preeclamptic pregnancies. However, we found that circulating levels of the proangiogenic proteins, VEGF and PlGF, were lower in the P5-PELS (Figure 4C and 4E), independently of their sex (Figure S7A and S7B). Additionally, translational evidence from fetal plasma showed a trend toward lower PlGF (*P*=0.09) but no changes in VEGF circulating levels (Figure 4D and 4F).

**Figure 5:**
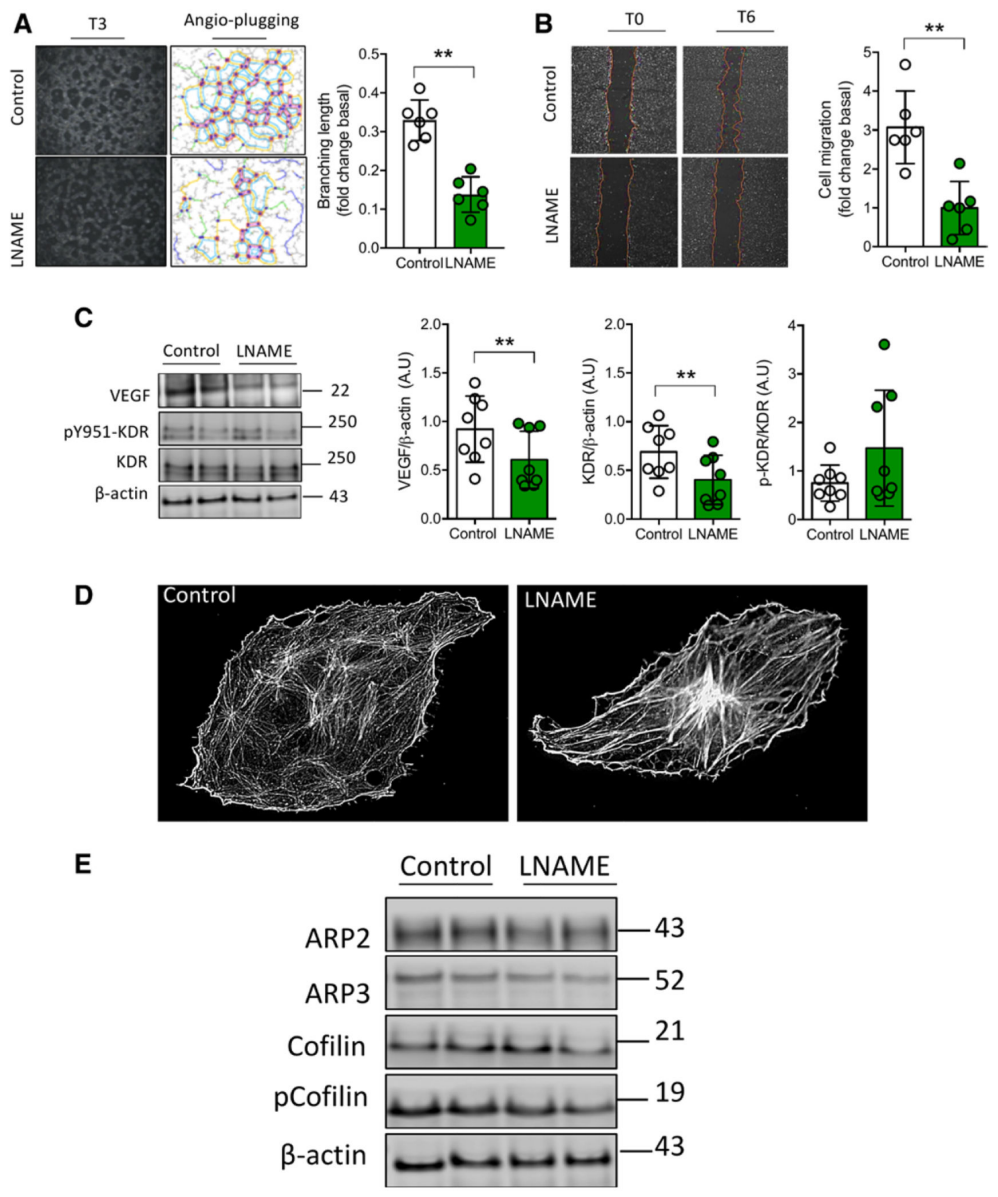
A circulating factor of postnatal day 5 (P5) preeclampsia-like syndrome (PELS) reduces in vitro angiogenesis and impairs F-actin polymerization. **A**, Representative image of in vitro angiogenesis in mice brain endothelial cells (Bend3) exposed to serum from P5-PELS (NG-nitroarginine methyl ester hydrochloride [L-NAME]; 3 h; 1%; v/v), showing reduced branching length in the L-NAME group. **B**, Representative images of cell migration (ie, scratch assay) in Bend3 treated with serum from the experimental groups (6 h; 1%; v/v), showing reduced cell migration after exposition to serum from P5 pups of L-NAME dams. **C**, Representative blots of VEGF (vascular endothelial growth factor), KDR (kinase insert domain-containing receptor), and tyrosine 951 phosphorylation of KDR (pY951-KDR) and respective densitometry. **D**, Representative image of F-actin visualization using the fluorescent dye phalloidin in Bend3 treated as in **C. E**, Representative blots of protein regulators of F-actin polymerization: ARP2/3 (actin-related proteins 2 and 3), cofilin (active form), or phosphorylated cofilin (inactive form) in cells treated as in **C**. β-Actin was used as a loading control in all blots. Each dot represents 1 study subject. Values are presented in the median±interquartile range. **P<0.005.

Furthermore, we exposed mice brain microvascular endothelial cell line (Bend3) isolated from a mouse brain with endothelioma and used previously in brain angiogenesis and neuroscience research,^41^ to serum from P5-PELS (L-NAME model) and control groups (Figure 5). First, we confirmed that serum from P5-PELS did not affect cell viability, as estimated by mitochondrial dehydrogenase activity (Figure S8A). However, serum from P5-PELS reduced cell proliferation (Figure S8B). We then observed a significant reduction in in vitro angiogenesis (in about 60%; *P*<0.05), branching length (Figure 5A), and number of junctions (Figure S8C; n=6 per group; *P*<0.05) in cells exposed to serum from P5-PELS. This reduction was associated with diminished cell migration in the presence of serum from P5-PELS (Figure 5B). We also conducted confirmatory experiments showing reduced cell migration between 3 and 24 hours of experimentation in the P5-PELS group (data not shown). Additionally, serum from P5-PELS reduced protein levels of VEGF, and KDR in Bend3 cells, compared with cells exposed to serum from P5 control mice. However, there were no changes in the 951-tyrosine phosphorylation of KDR (Figure 5C) or in the VEGF released from Bend3 cells (Figure S8D and S8E) exposed to the serum of P5-PELS.

### Brain Endothelial Cell Migration After Exposure to Offspring Serum From Preeclampsia

To further explore alterations in brain endothelial cell migration, we evaluated the length and width of F-actin filaments and filopodia assembly in Bend3 cells (Figure 5D). Serum from P5-PELS generated an increase in the filament length of F-actin (0.011±0.003 versus 0.006±0.003 length fibers per area of the cell, respectively; n=10–13; *P*=0.02), without changes in the filament width compared with cells exposed to serum from control pups. Additionally, a significant reduction in the filopodia number was found in Bend3 cells treated with the serum of P5-PELS (138±55 versus 223±41 filopodium per cell, respectively; n=6; *P*=0.02). These findings were associated with a reduction in both ARP2 (*P*=0.02) and ARP3 complex (Figure 5E; n=8 per group; *P*=0.001), without changes in cofilin or phosphorylated cofilin (Figure 5E) in Bend3 cells exposed to the serum of P5-PELS.

**Figure 6:**
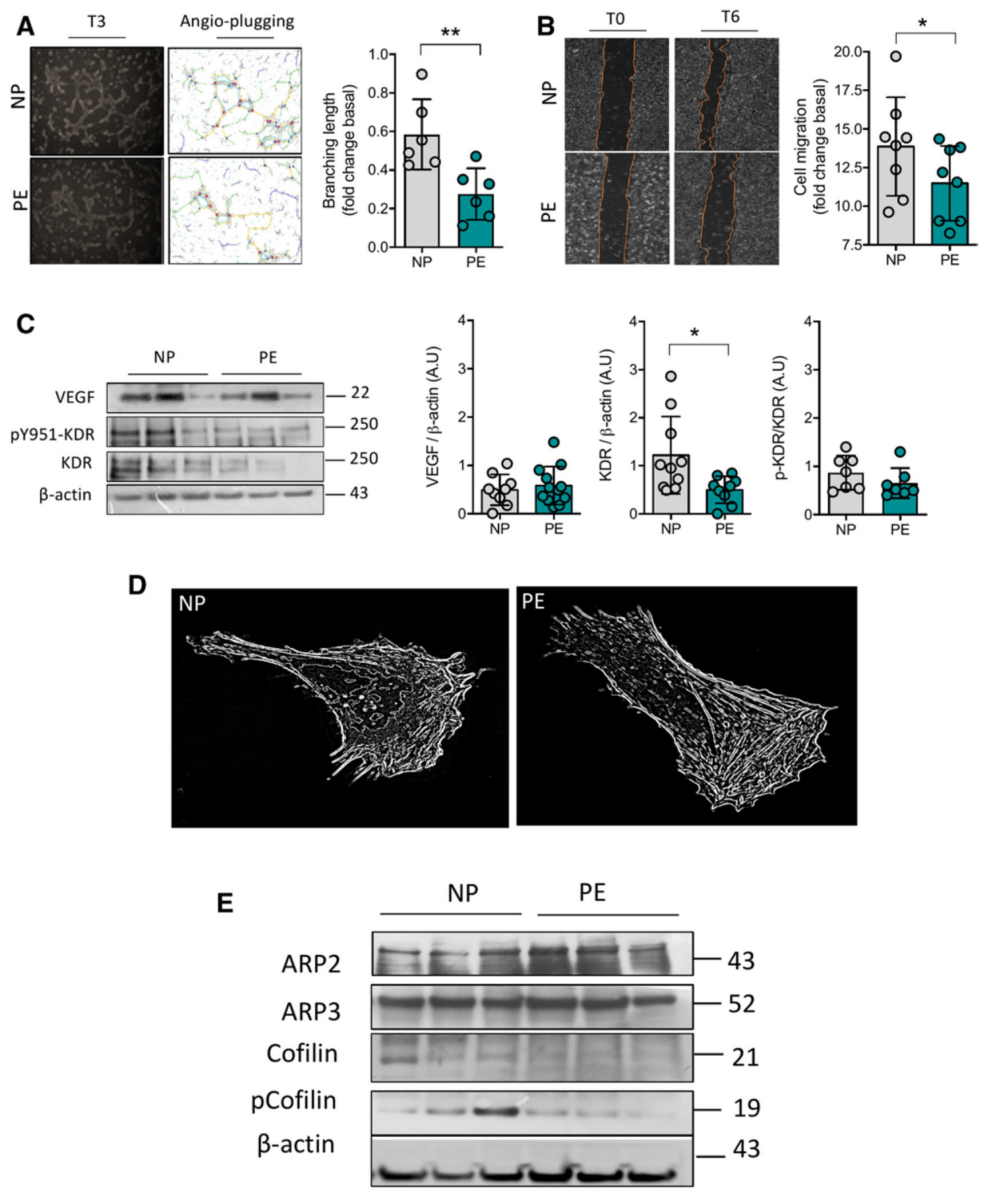
Circulating factor present in plasma from fetuses of women with preeclampsia reduces in vitro angiogenesis and impairs F-actin polymerization. **A**, Representative image of in vitro angiogenesis in human brain endothelial cells (hCMEC/D3) exposed to umbilical cord plasma (fetal) from newborns of women with normal pregnancy (NP) or women with preeclampsia (PE; 3 h; 1%; v/v), showing reduced branching length (ie, in vitro angiogenesis) in cells exposed to fetal plasma from PE pregnancies. **B**, Representative images of cell migration (ie, scratch assay) in hCMEC/D3 treated with the fetal plasma (PE; 6 h; 1%; v/v), showing reduced cell migration in cells exposed to PE fetal plasma. **C**, Representative blots of VEGF (vascular endothelial growth factor), KDR (kinase insert domain-containing receptor), and tyrosine 951 phosphorylation of KDR (pY951-KDR) and respective densitometry analysis. **D**, Representative image of F-actin visualization using the fluorescent dye phalloidin in hCMEC/D3 cells treated as in **C. E**, Representative blots of protein regulators of F-actin polymerization: ARP2/3 (actin-related proteins 2 and 3), cofilin (active form), or phosphorylated cofilin (inactive form) in cells treated as in **C**. *β*-actin was used as a loading control in all blots. Each dot represents 1 study subject. Values are presented in the median±interquartile range. *P<0.05, **P<0.005.

To translate these findings to the human setting, we used the human cerebral microvasculature endothelial cell line hCMEC/D3 and exposed them to fetal plasma from women with preeclampsia. Results were similar to what we observed in mouse brain endothelial cells, showing no changes in cell viability in cells exposed to fetal plasma (Figure S9A). Notably, hCMEC/D3 exposed to fetal plasma from preeclamptic pregnancies showed reduced cell proliferation (Figure S9B), in vitro angiogenic capacity (Figure 6A; Figure S9C), as well as cell migration (Figure 6B), compared with those exposed to plasma of normotensive controls. Reduced protein levels of KDR, without changes in the synthesis (Figure 6C) or release of VEGF (Figure S9D and S9E) or in the 951-tyrosine phosphorylation of KDR (Figure 6C), were also found in hCMEC/D3 cells exposed to fetal plasma from preeclampsia. Similar to our observations in Bend3 exposed to serum from P5-PELS, we observed alterations in the F-actin filaments, characterized by increased fiber length (0.04±0.02 versus 0.03±0.01 length fibers per area of the cell, respectively; n=16–20; *P*=0.02). However, fetal plasma from preeclampsia reduced fiber width (*P*<0.0001; Figure 6D) and filopodia number (87.8±51.2 versus 189.4±115.3 filopodium per cell, respectively; n=7; *P*=0.03) in hCMEC/D3. These alterations in the F-actin fibers were associated with reduced protein levels of cofilin (n=8 per group; *P*=0.02). However, contrary to what we found in mouse brain endothelial cells, in human cells, we did not find significant changes in the protein levels of the ARP2/3 complex (Figure 6E) after exposure to the fetal plasma from preeclampsia.

## Discussion

This study presents the first evidence of reduced brain angiogenesis in offspring (P5) of a mouse model of preeclampsia. Potential underlying mechanisms contributing to these results include reduced brain endothelial cell migration, alterations in the VEGF/VEGFR signaling, and disassembling of actin filaments. These alterations may predispose to significant consequences on the downstream cognitive abilities of the offspring, as suggested by others.^42,43^

### Brain Vascular Alterations in Offspring From Preeclampsia

For the first time, we describe that P5-PELS (from 3 different preeclampsia models) exhibits reduced brain angiogenesis compared with their counterparts in the control group at the same neonatal age (Table S3). This may explain our previous findings of reduced cerebral blood flow observed in the motor and somatosensory cortex of P5-PELS.^10^ Furthermore, these findings agree with previous reports^12,13^ showing impaired brain vascular function in both the middle cerebral artery and vein of Galen after birth (postnatal days 16–30) in off-spring from PELS. Nevertheless, our results contrast with another study in adult offspring (12 months of age) of PELS generated in transgenic female mice over-expressing human angiotensinogen and renin, which showed increased cerebral blood flow in response to whisker stimulation.^44^ The apparent disparity between these findings may be associated with differences in the PELS model used to study the offspring. In this regard, to overcome the potential bias generated using L-NAME, we confirmed the finding of reduced brain angiogenesis in 2 additional mouse models, the RUPP model^10^ and the genetic C1q model of preeclampsia.^39^ It is unclear whether this reduction in the brain angiogenesis has functional implications in the offspring; however, previous findings using offspring from L-NAME–treated pregnant rats^45^ or in mice in which alterations in brain angiogenesis were due to 16p11.2 deletion (as a model of autism),^46^ or deletion of PlGF,^47^ have demonstrated severe behavioral and cognitive alterations in the offspring.

We explore the underlying mechanisms of reduced brain angiogenesis in P5-PELS, reporting low VEGF and PlGF circulating levels. Reduced VEGF and PlGF may profoundly affect brain vascular development and neurodevelopment.^14,45,48^ Accordingly, we report a low relative VEGF and PlGF trend in human fetal plasma of preeclampsia, which agrees with other studies using quantitative ELISA assays, showing significantly reduced VEGF,^49,50^ or PlGF^50^ levels in fetal serum from preeclampsia. Nevertheless, like other studies using fetal serum,^49,51^ we report no differences in the circulating levels of sFLT1, neither in the analyzed P5-PELS nor human fetal plasma from preeclampsia. Therefore, we hypothesize that reduced circulating levels of VEGF and PlGF, without changes in the circulating sFLT1 levels observed in P5-PELS, may generate an impaired VEGFR brain endothelial signaling regulating angiogenesis. However, although relevant and novel, we acknowledge that our findings do not determine causality. We also present novel evidence showing reduced VEGFR2 (or KDR) in brain endothelial cells exposed to the serum of P5-PELS or fetal plasma from preeclamptic pregnancies. Therefore, these results suggest an imbalance between circulating VEGF/PlGF and sFLT1 present in preeclampsia offspring may reduce KDR-dependent brain angiogenesis. Accordingly, reduced cell migration and proliferation were found in brain endothelial cells (mice and humans) exposed to serum/plasma from off-spring of preeclampsia; however, how these cellular outcomes might relate to the phosphorylation of the KDR warrants further investigation. Because cell migration is critical to brain angiogenesis at the early postnatal age in mice,^24,33^ and KDR has been linked with VEGF-mediated cytoskeletal reorganization,^52^ we report alterations in F-actin polymerization. Very little is known about alterations in the cytoskeleton in fetal endothelial cells. Only 1 prior report described some filament alterations, characterized by an increased number of intermediate filaments that occupied the cytoplasm in placental endothelial cells of women with preeclampsia.^53^ Compatible with our findings, a previous report has shown increased phosphorylated cofilin levels in trophoblast cell lysates from women with preeclampsia compared with controls. This leads to disarrangement of the cyto-skeleton and loss of spatial orientation of F-actin fibers, possibly impairing receptor recycling and degradation.^54^ Importantly, the Arp2/3 complex is a crucial regulator of lamellipodia and filopodia protrusion formation by initiating branched F-actin polymerization,^55^ which has not been studied in the context of preeclampsia. Regulation of the actin cytoskeleton is essential for cell adhesion maintenance and formation of lamellipodia and filopodia protrusions, contributing to the tightness of the blood-brain barrier.^56^ Whether the changes in F-actin and filopodia observed in this study may also affect other functions of the brain endothelial cells, such as the tightness of cell-to-cell interaction to form the blood-brain barrier, is unknown. It has been shown, however, that increased leakiness of the in vitro model of the blood-brain barrier, using human brain endothelial cells, was associated with a reduction in the Rac1 (ras-related C3 botulinum toxin substrate 1)/WAVE (Wiskoff-Aldrich Syndrome protein-family verprolin homologous protein)/Arp3 (actin related protein 3) signaling pathway.^57^ Our findings suggest the presence of echographic signs of brain edema in P5/PELS^10^ as a raw indicator of blood-brain barrier disruption. We will pursue future studies to further investigate the cytoskeletal alterations in brain endothelial cells as triggers of impaired vessel formation and function in preeclampsia.

Based on our previous findings in the RUPP model^10^ and our current observations, we propose that placental dysfunction may release potential deleterious factors toward the fetal circulation, as described on the maternal side.^58^ Supporting this hypothesis, our P5-PELS model had reduced placental efficiency (fetal weight/placental weight ratio), suggesting that placental malperfusion and subsequent hypoxia generate a less efficient placenta regarding oxygen and nutrient transport toward the fetus. This idea has been postulated in an emerging area of research called neuroplacentology.^59^ However, it is unclear how placental dysfunction may shape brain development. Among the various potential placental circulating factors, in this study, we propose that an imbalance between circulating VEGF/PlGF/sFLT1 in P5-PELS may contribute to the observed reduced brain angiogenesis.

We acknowledge some limitations in our study. For example, we and others^7–9,45^ have used L-NAME administration as a PELS model to study brain complications in the offspring of preeclampsia. Because preeclampsia is a human condition, every single model has limitations. Brain angiogenesis was studied in 2 additional pre-eclampsia models to overcome the potential limitation of the L-NAME model. Nevertheless, because infants born from preeclamptic pregnancies already have a series of comorbidities associated with adverse perinatal outcomes,^60^ it is unsurprising that male babies may exhibit a higher rate of brain complications than their female counterparts. However, this relationship needs to be better delineated in the literature. Some animal studies have demonstrated a higher predisposition to cerebral complications in male than female offspring of preeclampsia.^61,62^ A properly designed study to specifically analyze potential sexual dimorphism in the brain angiogenesis formation and function in offspring from preeclampsia is underway in our laboratory.

In conclusion, our study demonstrates that offspring of preeclampsia exhibit a reduction in brain cortex angiogenesis, which is likely contributed by reduced levels of circulating VEGF/PlGF/KDR proteins. These alterations may lead to impaired brain endothelial migration due to the dysfunctional disassembly of F-actin filaments, potentially resulting in structural and functional alterations in brain development.

### Perspectives

Our findings indicate that reduced brain blood perfusion observed in children of mothers with preeclampsia^11^ and pups of PELS^10^ is likely associated with reduced brain cortex angiogenesis. These findings contribute to our understanding of structural, behavioral, and cognitive alterations described in children born from women with preeclampsia.^4–6^

A key question is when these alterations in brain angiogenesis begin. In severe preeclampsia, the fetus redistributes its cardiac output to maximize oxygen and nutrient supply to the brain, a phenomenon called fetal brain sparing or preferential cerebral perfusion in response to placental insufficiency.^63^ This redistribution of fetal blood flow to the brain is a compensatory mechanism protecting the fetus from potential damage.^64^ Despite that, epidemiological data show that brains that have been spared are nevertheless compromised and associated with elevated risks for brain-associated adverse outcomes, including compromised cognitive function. Thus, brain angiogenesis alterations likely start during the intrauterine period and persist immediately (days) after birth,^65^ constituting a critical developmental window. Notwithstanding these findings, we wish to make clear that fetal brain blood flow alterations intrauterine or immediately after birth are not exclusive to preeclampsia because they are also observed in fetal growth restriction, postterm pregnancies, previous pregnancy loss, and women with hypertension, diabetes, or other maternal pathology.^66^ Therefore, the outcomes demonstrated in the study will need to be further explored before drawing clinical conclusions.

## Supplementary Material

Supplementary Material

## Data Availability

The data supporting this study’s findings are available from the corresponding author upon reasonable request. Also, the Supplemental Material and its respective references^10,26–39^ include details of methods. Figure S1 summarizes the experimental diagram and timelines of the experimental models.

## Novelty and relevance

### What Is New?

We report that reduced angiogenesis in the brain cortex of offspring from preeclampsia-like models is likely contributed by reduced circulating VEGF (vascular endothelial growth factor)/PlGF (placental growth factor)/KDR (kinase insert domain-containing receptor) proteins. These alterations may lead to impaired brain endothelial migration due to the dysfunctional disassembly of F-actin filaments.

### What Is Relevant?

This work helps to understand the potential role of impaired brain angiogenesis in the structural alterations of brain development observed in children born from women with preeclampsia.

### Clinical/Pathophysiological Implications?

Our study demonstrates that offspring of preeclampsia exhibit a reduction in brain cortex angiogenesis, which likely involves reduced circulating levels of VEGF/PlGF/KDR and impaired brain endothelial migration due to the dysfunctional disassembly of F-actin filaments. These alterations may predispose to structural and functional alterations in long-term brain development observed in children born from preeclamptic pregnancies.

## Nonstandard Abbreviations and Acronyms

ARP2/3: actin-related proteins 2 and 3
KDR: kinase insert domain-containing receptor
L-NAME: NG-nitroarginine methyl ester hydrochloride
P5: postnatal day 5
PELS: preeclampsia-like mouse model
PlGF: placental growth factor
RUPP: reduction of uterine perfusion
sFLT1: soluble fms-like tyrosine kinase
VEGF: vascular endothelial growth factor
VEGFR: vascular endothelial growth factor receptor
