# Supplementary material for: Reduced Brain Cortex Angiogenesis in the Offspring of the Preeclampsia-Like Syndrome
Source: Hypertension. Author manuscript; Available in PMC 2025 Sep 4. (PMC7618073; doi:10.1161/HYPERTENSIONAHA.123.21756)
Supplement: Supplementary Material [file EMS208251-supplement-Supplementary_Material.zip › hyp_hype-2023-21756_supp3.pdf]

*** Short In Vivo Checklist**

*AHA - Preclinical Animal Testing : Prevention of bias is important for experimental cardiovascular research. **This short checklist must be completed, and the answers should be clearly presented in the manuscript as well.** The checklist will be used by reviewers and editors but will not be published. If a revision is requested, you will be required to complete at revision submission a more detailed checklist that will be published with the accepted article.*

This study involves animals:
Yes

Animals

Species, age, sex, strains, and sources of animals are described:	Yes

Randomization

Randomization and allocation concealment were performed:	Yes

Blinding

Blinding was performed:	Yes

Inclusions and Exclusions (a)

Specific criteria for inclusions and exclusions are specified:	N/A

Inclusions and Exclusions (b)

Criteria for inclusions and exclusions were set before the study:	N/A

Reporting of Excluded Animals

All animals excluded after the randomization are reported:	N/A

Statistical Methods

Statistical Methods are described:	Yes

Date completed: 08/22/2023 21:25:04
User pid: 267271
