# Supplementary material for: Reduced Brain Cortex Angiogenesis in the Offspring of the Preeclampsia-Like Syndrome
Source: Hypertension. Author manuscript; Available in PMC 2025 Sep 4. (PMC7618073; doi:10.1161/HYPERTENSIONAHA.123.21756)
Supplement: Supplementary Material [file EMS208251-supplement-Supplementary_Material.zip › hyp_hype-2023-21756_supp1.docx]

**Reduced brain cortex angiogenesis in the offspring of preeclampsia-like syndrome.**

Felipe Troncoso^1^, Hermes Sandoval^1^, Belén Ibanez^1^, Daniela López-Espíndola^2,3^, Francisca Bustos^2^, Juan Carlos Tapia^4^, Pedro Sandaña^5^, Esthefanny Escudero-Guevara^1^, Francisco Nualart^6,7^, Eder Ramírez^6^, Robert Powers^8^, Manu Vatish^9^, Hiten D. Mistry^10^, Lesia O. Kurlak^11^, Jesenia Acurio^1^, Carlos Escudero^1,3^.

*^1^Vascular Physiology Laboratory, Department of Basic Sciences, Universidad del Bío-Bío, Chillán, Chile.*

*^2^ Escuela de Tecnología Médica, Facultad de Medicina, Universidad de Valparaíso, Valparaiso, Chile.*

*^3^ Group of Research and Innovation in Vascular Health (GRIVAS Health), Chillan, Chile.*

*^4^ Stem Cells and Neuroscience Center, School of Medicine, University of Talca, Campus Talca, Talca, Chile.*

*^5^ Anatomopatholy Unit, Hospital Clinico Herminda Martin, Chillan, Chile*

*^6^ Laboratory of Neurobiology and Stem Cells NeuroCellT, Department of Cellular Biology, Center for Advanced Microscopy CMA BIO BIO, Faculty of Biological Sciences, University of Concepcion, Concepcion, Chile.*

*^7^ Departamento de Biología Celular, Facultad de Ciencias Biológicas, Universidad de Concepción, Casilla 160-C, Concepción, Chile.*

*^8^ Magee-Womens Research Institute, Department of Obstetrics, Gynecology and Reproductive Sciences, University of Pittsburgh, Pittsburgh, PA, USA.*

*^9^ Nuffield Department of Women's Health and Reproductive Research, University of Oxford, Oxford.*

*^10^ Division of Women and Children's Health, School of Life Course and Population Sciences, King's College London, London, UK.*

*^11^ Stroke Trials Unit (School of Medicine), University of Nottingham, Nottingham, UK.*

*Correspondence: Carlos Escudero, MD PhD

Vascular Physiology Laboratory

Group of Research and Innovation in Vascular Health

Basic Sciences Department

Faculty of Sciences

Universidad del Bio-Bio

Chillán, Chile

Phone: 56-42-2463256 / Mobile: 56-9-65655127

cescudero@ubiobio.cl

**Running title:** Brain angiogenesis in offspring from preeclampsia

**Manuscript word count: 7606**

**Abstract word count: 249**

**Financial disclosure:** Fondecyt 1200250, 1200951

**Conflict of interest:** none

**Number of figures:** 6

**The number of references:** 66

**Supplementary information:** Table S1, S2, S3. Figure S1-S9

**Keycode:** brain angiogenesis, offspring, and preeclampsia.

**Extended methods**

***Human samples***

We included random umbilical cord venous (fetal) plasma samples from 21 normotensive control pregnant women and 19 pregnant women with preeclampsia from our biobank. The study was conducted according to the guidelines of the Declaration of Helsinki and approved by the HRA-REC ethics committee of the University of Nottingham (REF: 15/EM/0523); written, informed consent was obtained from each participant. The clinical diagnosis of preeclampsia was defined as systolic blood pressure ≥140 mmHg and diastolic (Korotkoff) pressure ≥90 mmHg (pressures taken at two different times after 20 weeks of gestation), and proteinuria >300 mg/L. In addition, medical and obstetric history data were obtained. The percentile weight of each baby at birth was corrected for gestational age. We included maternal parity and calculated body mass index (BMI). Umbilical (fetal) venous blood samples were drawn immediately after delivery and stored in aliquots of refrigerated tubes containing EDTA at -80°C. Samples were shipped to Chile at the Vascular Physiology Laboratory at the University of Bio Bio to conduct further experiments using human brain endothelial cells.

***Preeclampsia-like models and experimental sample size***

The Bioethics and Biosafety Committee of the Universidad del Bío-Bío approved the animal management and supervision protocols used in this study in agreement with the Guide for Care and Use of Laboratory Animals guidelines^1^ and 3R principles referring to humanized use of animals. All experiments are reported following the ARRIVE guidelines 2.0 (Animal Research: Reporting *in Vivo* Experiments) for how to report animal experiments. Female and male C57BL/6 mice were housed in a 25°C temperature and humidity-controlled room in the Vivarium belonging to Universidad del Bío-Bío. Inbred strains of mice obtained in our animal facility were used for all experiments. Light–dark cycles were 12:12 hours, and mice were fed with balanced food (Prolab RMH 3000, Labdiet, St. Luis, Missouri, USA) and water ad-libitum, as previously described in our laboratory^2, 3^.

At 3–5 months, female mice crossed with C57BL/6 male mice of similar age. The presence of a vaginal plug was designated as gestational day 0 (D0). Three different animal models of preeclampsia were used. First, wild-type C57BL/6 pregnant mice were supplemented in drinking water with the nitric oxide synthase inhibitor, NG-Nitroarginine methyl ester hydrochloride, L-NAME (150 mg/kg/d) since D7 of gestation, as previously described^4^, with minor modifications. Pregnant mice were sacrificed at gestational day 19 (D19) for preeclampsia-like syndrome characterization. In this model, twenty pregnant mice were included. They were randomly assigned to L-NAME (n=10 dams) or control (n=10 dams) groups. Two experimental windows were included, at D19 of gestation (n= 5 per group) and postnatally P5 (n=5 per group). From these dams, 116 pups were included, 60 in the control group (n=37 at D19 and n=23 at P5); and 56 in the L-NAME group (n=37 at D19 and n=19 at P5).

A second model of preeclampsia was generated using the reduction of uterine perfusion (RUPP), as we have previously described^2, 3^. Briefly, C57BL/6 pregnant mice at 14.5 dpc (Sham, n=3; RUPP, n=3) were anesthetized with isoflurane 3% before laparotomy performed on a thermoregulated platform (Kent Scientific, Torrington, Connecticut, USA). Surgical exposition of the uterine horns allowed the analysis of uterine blood vessels and the presence of embryos. In the RUPP group, uterine arterial and ovary arteries were partially cauterized on both sides. Analgesia post-surgery was conducted using ketoprofen (5 mg/kg, subcutaneously) every 24 hours for three days. From this model, three pups (P5) per litter were randomly chosen in each experimental group (N=9 pups per group).

Lastly, a unique genetic model of preeclampsia originally published by Singh et al.^5^ and further investigated by Sutton et al^6^ was utilized.  The C1q model has been shown to recapitulate the features of preeclampsia including: pregnancy specific hypertension, albuminuria, endotheliosis, vascular dysfunction, lower placental VEGF, elevated sFlt-1, and elevated fetal death. In this model, eight-week-old female C57BL/6J mice (Jackson) were time-mated to either C57BL/6J or C1q^−/−^ male mice to model an uncomplicated pregnancy (WT, n=5 dams) or preeclampsia-like pregnancy (PE, n=5 dams), respectively. In addition, a third comparative group was generated in which C1q−/− female mice were time-mated to wild-type males (genetic control, GC, n=4 dams). The brains of the offspring were collected following the previously validated protocol^7^. We analyzed 35 pups' brains from this model, divided into the three experimental groups (WT, n=13; GC, n=12; and PE, n=10 pups).

Using C57BL/6 mice in all preeclampsia-like models, we prevented potential strain-dependent differences. In addition, immunohistochemistry was performed in a different laboratory in a blind manner of their respective experimental groups.

***Protein quantification, western blot, and ELISA***

Protein extraction from different sources, serum in mice, plasma in umbilical cord human samples (fetal), brain tissue homogenate, and mice and human brain endothelial cell extraction used similar approaches of western blot, as we described previously^2, 8^. Supplementary Table S1 details the used antibodies. The ELISA assay quantification of circulating sFLT-1 was performed according to the manufacturer's protocol (R&D Systems, Minneapolis, Minnesota, USA). Fetal plasma from the human and serum from D19 dams or P5 pups were used. Briefly, fetal plasma from the human and serum from D19 dams or P5 pups were diluted in diluting buffer provided with the kit in a 1:1 v/v ratio. First, 100 μL of diluent, then 100 μL of the standard curve, and the respective plasmas were added to each well and allowed to incubate for 2 hours at room temperature with shaking. After washes, 200 µl of Flt-1 antibody (VEGFR1) are added and incubated for 2 hours at room temperature. After that, 200 μl of substrate solution was added for 30 minutes at room temperature. After the time has elapsed, the blocking solution is added for 30 minutes and read with an absorbance reader (Biotek, microplate reader 800 TS) at a wavelength of 540 nm.

***Histology – Analysis of kidney, placenta, and aorta histology samples.***

On D19, placentae, kidney, and aorta samples (and offspring, see below) were removed for histological characterization using hematoxylin and eosin staining. Tissues were fixed in paraformaldehyde (4% in buffer phosphate, v/v) for 24 hours and subsequently embedded in paraffin for histological analysis and immunohistochemistry. Each sample was cut (3 µm thick) into multiple sections with a Multicut 2045 microtome (Leica, Allendale, New Jersey, USA) and stained with hematoxylin-eosin. For the observation and analysis of each section, a Motic BA410 microscope (Motic, HK, CHN) and the Image J program (National Institutes of Health, NIH) were used. In the kidney, 5 random sections of the kidney section and 3 glomeruli of each were analyzed. The total glomerular, mesangial, and glomerular capillary areas were also quantified^9^. In the placenta, the areas of the maternal decidua, functional area, and labyrinth area were analyzed, the percentages were obtained considering the total area of the placenta, and placental efficiency was calculated as the ratio of fetal body weight/placental weight^10^. In aortas, the total area and perimeter, the width in 5 random zones, and the number of endothelial cells in a delimited zone (1 arbitrary unit) were measured.

***Offspring morphometry and measurements***

Similarly, at D19 and postnatal day 5 (P5), morphometric parameters were registered in pups, including weight and size (determined by cephalo-caudal length). Pups were sexed by visual evaluation and confirmed by PCR using the expression of the jarid gene, as we previously described^8^. All analyses of the pups included one to three males and females per litter in each group.

***Cortex brain blood vessel immunohistochemistry***

We selected the motor and somatosensory cortex to identify brain blood vessels with or without a functional lumen. Angiogenesis markers in brain tissue were measured in different laboratories and were blinded to the outcome group. Then, P5 pups were perfused with Evan's Blue (Sigma-Aldrich, Mo, USA)^7^ or vascular selective lectins prepared with an anti-laminin antibody produced in rabbits (Sigma-Aldrich, Mo, USA). Those fluorescent dyes were injected either via intraocular or intracardiac injection, respectively. After perfusion, Evan's blue injected brains were fixed in paraformaldehyde (4% in PBS, v/v, 24 h) and then embedded in paraffin. In contrast, mice injected with vascular selective lectin were fixed in paraformaldehyde and directly cut using a vibratome.

In addition, for immunostaining of endothelium, we used the *Bandeiraea simplicifolia* BS-I isolectin B4^7^ (IBA4, Sigma-Aldrich, Mo, USA) or GLUT1 immunostaining^11^. We used a confocal microscope (Zeiss 780 LMD, Germany) for fluorescent tracer visualization. The AngioTool software was used for confocal analysis^12^, in which vessel area, the total number of vascular junctions, and total vessel length was estimated using the image processing algorithm consisting of image transformation to 16-bit grayscale and subsequent vessel identification by segmentation and skeletonization analysis. Also, a bright-field microscope was used for IB4 staining observation. The intensity of IB4 staining was analyzed in Image J software as described previously^13^, in which the immunostained signal was extracted from the images with a color deconvolution algorithm and expressed by the total area of the image. We estimated the total number of blood vessels (i.e., GLUT1 or IB4 positive) and the vessels with a functional lumen (i.e., positive to lectin or Evan’s blue) named vascularized vessels in the brain cortex of P5-PELS.

***Brain endothelial cell cultures.***

Human and brain microvascular endothelial cells were used. For the human, the commercial line of human cerebral microvasculature endothelium hCMEC/D3 (Millipore, CA, USA) was used following manufacture instructions as we have described previously^14^. Briefly, cells were cultured using an EndoGroTM growth medium (Millipore, CA, USA) and enriched with an EndoGroMV kit (Millipore, CA, USA), whose content includes ascorbic acid 50 μg/ml; heparan sulfate 50 U/ml; hydrocortisone 1 μg/ml; 10 mM L-glutamine; 5 ng/ml EGF, 5% fetal bovine serum (FBS), and 1% penicillin-streptomycin (10,000 U/ml) (Gibco, NY, USA). To generate adhesion to the culture plates, they were pretreated with a type I collagen matrix (Sigma, MO, USA) for 30 minutes under controlled conditions (37°C and 5% atmospheric CO2).

In addition, the mouse brain endothelium line bEnd3 was also used in selected experiments following manufacture instructions (Sigma-Aldrich, Mo, USA). Briefly, cells were cultured using DMEM growth medium (Gibco, NY, USA) containing 2mM Glutamine, 5μM 2-Mercaptoethanol (2ME), 1mM Sodium Pyruvate (NaP), 1% Non-Essential Amino Acids (NEAA), 10 % Fetal Bovine Serum (FBS), which was supplemented with fetal bovine serum (FBS) and 1% penicillin-streptomycin (10,000 U/ml) (Gibco, NY, USA). The culture plates were pretreated with a type I collagen matrix (Sigma, MO, USA) under controlled conditions (37ºC and 5% atmospheric CO2). Both kinds of cells were used at 8 to 12 passages. Before the experiments, the cells were deprived overnight of serum and growth media to synchronize their function.

***Viability by MTT assay***

Viability assays were performed using the CellTiter 96 Non-Radioactive Kit (Lot: 0000105232, Promega, USA). Human or mouse brain endothelial cells were treated (12 h, 1% v/v) with the fetal plasma of women with normal pregnancies or preeclampsia; or serum from control and L-NAME pups (P5), respectively. Absorbance was analyzed using Epoch spectrophotometer equipment (BioTek Instruments, VT, USA), with an absorbance of 570 nm, as previously described^14^.

***Cell proliferation***

Cell proliferation was analyzed using 5-bromo-2-deoxyuridine (BrdU 10 mM), incorporation assay (Roche, INDY, USA), in brain endothelial cells (human and mice) treated (24 h) with umbilical cord plasma or serum from P5 pups as described above. The luminescence was quantified with Epoch spectrophotometer equipment (BioTek Instruments, VT, USA), with an absorbance of 540 nm.

***Cell migration***

Cell proliferation was analyzed using the *in vitro* “wound healing assay” in confluent brain endothelial cells (human and mice). Once confluent, cells were serum-deprived for 8 hours and then stimulated (0-6 h, 1% v/v) with umbilical cord plasma or serum from P5 pups, as described above. Then, a "scratch" was made with Scrather SPL Scar (Kisker, ST, DE) in a vertical position in each of the wells. The event is recorded by taking photographs with an Olympus Japan microscope and MShot MD90 camera (MShot Technology, GZ, CHN) every 3 hours until 12 hours, considering the initial time T=0. The images were quantified using the Image J v5.2 program (NIH, USA). The percentage of migration between the initial area T=0 and the area migrated at different times T=3, T=6 were calculated using the following formula, as described previously^8^.

| *M. area* = (*A*_0_-*A*_xh_/*A*_0_) * 100 |  |
| --- | --- |

Where *M. area* represented the migratory area, *A*_0_ represented the area at time 0, or the denudated area, and *A*_xh_ represented the area that remained denuded after 3 or 6 hours.

***In vitro angiogenesis***

Brain endothelial cells (4 × 10^4^) were cultured on a 96-well plate pre-coated with 40 μL Matrigel basement membrane matrix (Corning Labware, MA, USA). Cells were treated as described above using fetal plasma or serum from P5 pups (3 h, 1% v/v). Tubes were photographed using an inverted phase contrast microscope under 10× magnification (Olympus, Tokyo, Japan). The formation of networks (branches and the number of junctions) was quantified using the "Angiogenesis Analyzer" plugin from ImageJ 1.48 software, as previously reported^8, 15^.

***Conditioned medium***

Brain endothelial cells (human and mice) at 90% confluence were washed three times with phosphate buffer solution (PBS) and then cultured in a growth medium supplemented with 1% fetal bovine serum (FBS) for 24 h. The conditioned media (CM) was collected and stored in aliquots at -80°C until use for analysis of VEGF using Western blot.

***F-actin measurements***

F-Actin fibers were identified using a fluorescent dye phalloidin iFluor 488 (1:500 dilution ab176753, Abcam, Cambridge, UK) in brain endothelial cells (human and mice) treated as described above using umbilical cord plasma or serum from P5 pups (6 h, 1% v/v). Subsequently, the fluorescent dye phalloidin was removed, and samples were consecutively washed with phosphate buffer solution (3X, 5 min). Then, nuclear labeling (DAPI) was added in dilution (1:10,000 v/v) for 15 minutes. Finally, all samples were transferred onto a 26 x 76 mm slide (Knittel Glass, BS, DE) and covered by a glass coverslip supported by mounting fluid for subsequent visualization using fluorescent microscopy (Motic model BA410, Motic, HK, China). We captured images with 100 X magnifications and a resolution of 2580 x 1944-pixel per image. Three RGB chromatic channels (Red, Green, and Blue) are used, with their corresponding filters; (FITC) (Ex 330-380), (TRITC) (Ex 450-490), and (DAPI) (Ex 510-560). Then, captured fluorescent cell images were analyzed with the Image J program (National Institutes of Health, NIH) using the Mexican Hat filter in each analysis, which preserves high frequencies, thus highlighting the fibers. The plot profile function was used to determine the F-actin's width. The Ride Detection plugins were applied to analyze the number and length of fibers. Finally, using a similar approach, the number of filopodia was estimated, extracting the whole image of the cell and leaving only the filopodia visible.

***Statistical analysis***

Quantitative variables are presented as median ± interquartile range, whereas qualitative variables are presented in percentages considering their respective group. Considering data distribution, we used parametric or non-parametric tests, as appropriate, using the Shapiro-Wilk normality test. Then, we compared between groups by student’s t-test or Mann-Whitney test. Finally, we used a one-way ANOVA Test to analyze the three comparative groups in the genetic model of preeclampsia. In case of significant differences, we included a Bonferroni *posthoc* test. In the analysis of brain homogenates and *in vitro* studies, where experiments were performed head-by-head, a paired t-test was used. P<0.05 was considered a statistically significant difference. Data and statistical analyses were performed using the Microsoft Excel database and GraphPad Prism 6 (GraphPad Software, CA, USA).

**References in the supplementary information**

1. National Research Council (US) Committee for the Update of the Guide for the Care and Use of Laboratory Animals. Guide for the Care and Use of Laboratory Animals. In: T. N. A. C. R. f. b. N. I. o. Health, ed. *Guide for the Care and Use of Laboratory Animals* Washington (DC); 2011.

2. Lara E, Rivera N, Gonzalez-Bernal A, Rojas D, Lopez D, Rodriguez A and Escudero C. Abnormal cerebral microvascular perfusion and reactivity in female offspring of reduced uterine perfusion pressure (RUPP) mice model. *Journal of Cerebral Blood Flow and Metabolism*. 2022;42:2318-2332.

3. Cumsille P, Lara E, Verdugo-Hernandez P, Acurio J and Escudero C. A robust quantitative approach for laser speckle contrast imaging perfusion analysis revealed anomalies in the brain blood flow in offspring mice of preeclampsia. *Microvasc Res*. 2022;144:104418.

4. Burke SD, Zsengeller ZK, Khankin EV, Lo AS, Rajakumar A, DuPont JJ, McCurley A, Moss ME, Zhang D, Clark CD, Wang A, Seely EW, Kang PM, Stillman IE, Jaffe IZ and Karumanchi SA. Soluble fms-like tyrosine kinase 1 promotes angiotensin II sensitivity in preeclampsia. *J Clin Invest*. 2016;126:2561-74.

5. Singh J, Ahmed A and Girardi G. Role of complement component C1q in the onset of preeclampsia in mice. *Hypertension*. 2011;58:716-24.

6. Sutton EF, Gemmel M, Brands J, Gallaher MJ and Powers RW. Paternal deficiency of complement component C1q leads to a preeclampsia-like pregnancy in wild-type female mice and vascular adaptations postpartum. *Am J Physiol Regul Integr Comp Physiol*. 2020;318:R1047-R1057.

7. Walchli T, Mateos JM, Weinman O, Babic D, Regli L, Hoerstrup SP, Gerhardt H, Schwab ME and Vogel J. Quantitative assessment of angiogenesis, perfused blood vessels and endothelial tip cells in the postnatal mouse brain. *Nat Protoc*. 2015;10:53-74.

8. Troncoso F, Herlitz K, Acurio J, Aguayo C, Guevara K, Castro FO, Godoy AS, San Martin S and Escudero C. Advantages in Wound Healing Process in Female Mice Require Upregulation A2A-Mediated Angiogenesis under the Stimulation of 17beta-Estradiol. *Int J Mol Sci*. 2020;21.

9. Morita M, Mii A, Shimizu A, Yasuda F, Shoji J, Masuda Y, Ohashi R, Nagahama K, Kaneko T and Tsuruoka S. Glomerular endothelial cell injury and focal segmental glomerulosclerosis lesion in idiopathic membranous nephropathy. *PLoS One*. 2015;10:e0116700.

10. Natale BV, Mehta P, Vu P, Schweitzer C, Gustin K, Kotadia R and Natale DRC. Reduced Uteroplacental Perfusion Pressure (RUPP) causes altered trophoblast differentiation and pericyte reduction in the mouse placenta labyrinth. *Sci Rep*. 2018;8:17162.

11. Nualart F, Godoy A and Reinicke K. Expression of the hexose transporters GLUT1 and GLUT2 during the early development of the human brain. *Brain Res*. 1999;824:97-104.

12. Zudaire E, Gambardella L, Kurcz C and Vermeren S. A computational tool for quantitative analysis of vascular networks. *PLoS One*. 2011;6:e27385.

13. Escudero C, Celis C, Saez T, San Martin S, Valenzuela FJ, Aguayo C, Bertoglia P, Roberts JM and Acurio J. Increased placental angiogenesis in late and early onset pre-eclampsia is associated with differential activation of vascular endothelial growth factor receptor 2. *Placenta*. 2014;35:207-215.

14. Leon J, Acurio J, Bergman L, Lopez J, Karin Wikstrom A, Torres-Vergara P, Troncoso F, Castro FO, Vatish M and Escudero C. Disruption of the Blood-Brain Barrier by Extracellular Vesicles From Preeclampsia Plasma and Hypoxic Placentae: Attenuation by Magnesium Sulfate. *Hypertension*. 2021;78:1423-1433.

15. Troncoso F, Acurio J, Herlitz K, Aguayo C, Bertoglia P, Guzman-Gutierrez E, Loyola M, Gonzalez M, Rezgaoui M, Desoye G and Escudero C. Gestational diabetes mellitus is associated with increased pro-migratory activation of vascular endothelial growth factor receptor 2 and reduced expression of vascular endothelial growth factor receptor 1. *PLoS One*. 2017;12:e0182509.

**Supplementary tables**

**Table S1. List of antibodies used to evaluate brain endothelial function and angiogenesis.**

| Antibody | Code | Brand | Concentration  (ng/ml) |
| --- | --- | --- | --- |
| VEGF (C-1) | sc-7269 | Santa Cruz | 200 |
| sFlt-1 (D-2) | sc-271789 | Santa Cruz | 200 |
| KDR | #2472 | Cell Signaling Technology | 1:1000 v/v dilution* |
| KDR-P (Y951) | #2476 | Cell Signaling Technology | 1:1000 v/v dilution* |
| PLGF | ab196666 | Abcam | 1000 |
| Cofilin E-8 | sc-376476 | Santa Cruz | 200 |
| Cofilin phospho E-5 | sc-271921 | Santa Cruz | 200 |
| ARP2 E-2 | sc-137250 | Santa Cruz | 100 |
| ARP3 A-1 | sc-48344 | Santa Cruz | 100 |
| HIF1a 28b | sc-13515 | Santa Cruz | 200 |
| Glut1 | 07-1401 | Sigma-Aldrich | 1:1000 v/v dilution* |
| Bax B-9 | sc-7480 | Santa Cruz | 200 |
| Bcl-2 C-2 | sc-7382 | Santa Cruz | 200 |
| β-actin | A5441 | Sigma-Aldrich | 200 |

*No register information about stock concentration.

**Table S2. Clinical parameters of women with normal pregnancy and preeclampsia**

| Parameter | NC (n=21) | PE (n=19) |
| --- | --- | --- |
| Age (yrs) | 28.1 ± 7.2 | 31 ± 6.5 |
| Body mass index (BMI, Kg/m^2^) | 26.4 ± 5.5 | 24.8 ± 3.5 |
| Smoking status |  |  |
| Non-smoker | 9 (53) | 11 (65) |
| Smoker | 8 (47) | 6 (35) |
| Parity |  |  |
| Nulliparous | 11 (65) | 10 (59) |
| Multiparous | 6 (35) | 7 (41) |
| Max. systolic blood pressure (mmHg) | 114 ± 3.8 | 155 ± 3.8* |
| Max. diastolic blood pressure (mmHg) | 75 ± 2.1 | 97 ± 5.0* |
| Proteinuria (g/L), median (min, max) | - | 1.0 (0.3, 9,4) |
| Early-onset PE | - | 6 (35) |
| Cesarean section  Birthweight (g) | 4 (24)  3439 ± 498 | 13 (76) *  2993 ± 735 |
| Corrected birthweight centile | 27.9 (17.5, 66.8) | 35.1 (8.6, 76.3) |
| Male/Female newborn (n/n) | 12/9 | 12/7 |
| Placental weight (g) | 661 ± 196 | 688 ± 194 |
|  |  |  |

Data represented as means ± SD or median (QR) as appropriate, except for smoking status, parity and Caesarean sections, and early-onset PE, shown as numbers (percentage). NC, normotensive control; PE, preeclampsia; BMI, body mass index. *P<0.05 between normotensive controls and women with preeclampsia.

**Table S3. Overview of the main findings in brain angiogenesis alterations in offspring from preeclampsia.**

| Characteristics | L-NAME model | Genetic PE model | RUPP model |
| --- | --- | --- | --- |
| Placental efficiency | Reduced | Reduced^6^ | Reduced^2^ |
| Brain weight/body weight | Increased | No change | Increased^2^ |
| Brain angiogenesis | Reduced* | Reduced** | Reduced** |
| Brain vessels with functional lumen | Reduced | Reduced | No change |

*Evidenced by reduced area of brain blood vessels and junction number using fluorescent lectin and confocal microscopy. This finding is confirmed by a reduction in the total number of blood vessels identified by GLUT1 immunofluorescence and low protein expression of GLUT1 in brain angiogenesis homogenates using western blot.

** Evidenced by reduced IB4 staining in immunohistochemistry analysis.

**Supplementary Figures**

**Figure S1. Diagram of animal model and experimental approach. A)**Diagram of the PELS model generated by administration of L-NAME (150 mg/kg/d) between D7 and D19 of gestation in pregnant mice. Preeclampsia-like syndrome characterization and placental and fetal morphometry was done at D19 of gestation. **B)**Experimental approach using pups (5 days postnatal, P5) using three different models to analyze brain angiogenesis (i.e., new blood vessel formation) in the motor and somatosensory cortex areas. These models were complemented with analysis of pro-angiogenic parameters in vitro using mice (bEND/3) and human (hCMEC/D3) brain endothelial cells exposed to the serum of P5-PELS or controls or plasma of human umbilical vein from normal or preeclamptic pregnancies, respectively.

**Figure S2. Characterization of the preeclampsia-like model in pregnant mice treated with L-NAME.** A) Maternal weight in non-pregnant (non-preg) and pregnant mice treated (L-NAME preg) or not (Control-preg) with L-NAME (100 μg/kg/d). B) A trend (T-test, n=9, p=0.07) for elevated mean arterial pressure (MAP) was found in pregnant mice treated with L-NAME at D19 compared to D5, post and pre-administration of L-NAME, respectively. C) High sFLT-1, but D) reduced PLGF circulating levels in L-NAME dams. E) No changes in the total number of pups or the F) males or G) female pups after administration of L-NAME were found. H) L-NAME administration reduces placental efficiency (placental weight/fetal weight) in male but not in I) female pups. Each dot represents one study subject. Values are presented in the median ± interquartile range. *p<0.05; **p<0.005, ****p<0.0001. In A, †p<0.05 versus pregnant mice treated with L-NAME.

**Figure S3. Anthropometry of P5 pups born to preeclampsia-like model.** Reduced weight in P5 pups from L-NAME dams in both A) male and B) female P5 pups. Associated with C) reduced length in the whole group and in both D) male and E) female P5 pups from L-NAME dams. Each dot represents one study subject. Values are presented in the median ± interquartile range. **p<0.005, ****p<0.0001.

**Figure S4. Reduced brain endothelial cell marker in offspring from the preeclampsia-like model.** Reduced relative protein levels of GLUT-1 in the brain cortex of P5 pups born to preeclampsia-like model estimated by A) fluorescence, or B, C) western blots. B-actin was used as a loading control. Each dot represents one study subject. Values are presented in the median ± interquartile range. *p<0.05, ***p<0.001.

**Figure S5. Reduced brain angiogenesis in offspring from the preeclampsia-like model.** A) Representative images of Evan's blue (right) and IB4 staining (left) in motor (1A-1C) and somatosensory areas (2A-2B) in P5 pups born from preeclampsia-like model generated by the reduction in uterine perfusion (RUPP) or sham controls. B) Reduced total number of blood vessels (i.e., IB4 positive staining), C) without changes in the brain blood vessels with a functional lumen (i.e., Evan's blue positive) in P5 pups from RUPP dams. Each dot represents one study subject. Values are presented in the median ± interquartile range. *p<0.05.

**Figure S6. Overview of a genetic model of preeclampsia.** A) Genetic model of preeclampsia (PE) consisted of a crossbreed of wild-type (WT) female C57/B6J mice with C1q deficient (C1q-/-) male C57/B6J mice. Controls include crossbreeding between female and male WT and C1q-/- female with male WT. B) No differences were found in the total number of pups, either in C) male or D) female pups. E) Representative images of the pup's brains, showing F) no changes in the three experimental groups' brain weight/body weight ratio.

**Figure S7. Plasma levels of VEGF and PLGF in male and female offspring from the preeclampsia-like syndrome.** Reduced plasma levels of A) VEGF and B) PLGF were found in male (blue) and female (pink) pups from L-NAME dams.

**Figure S8. A circulating factor of P5 pups of preeclampsia-like syndrome reduces cell proliferation without affecting cell viability in mice brain endothelial cells.** A) No changes in cell viability marker estimated by MTT assay, but B) reduced cell proliferation was found in mice brain endothelial cells (Bend3) exposed to serum from P5 pups of L-NAME dams. C) In vitro angiogenesis assay showed reduced junctions in Bend3 exposed to serum from P5 pups of L-NAME dams. D) Representative image of VEGF present in the conditioned medium (CM) of Bend3 exposed (24 h, 1%, v/v) to serum from P5 pups of L-NAME dams. Ponceau staining was used as a loading control. E) No significant differences were found in VEGF levels in the CM between experimental groups (T-test, n=4 per group, p=0.17). Each dot represents one study subject. Values are presented in the median ± interquartile range. *p<0.05.

**Figure S9. Umbilical cord circulating factor from preeclampsia reduces cell proliferation without affecting cell viability in mice brain endothelial cells.** A) No changes in cell viability marker estimated by MTT assay, but B) reduced cell proliferation was found in human brain endothelial cells (hCMEC/D3) exposed to umbilical cord plasma from preeclamptic pregnancies. C) In vitro angiogenesis assay showed no significant differences in the number of junctions in hCMEC/D3 exposed to umbilical cord plasma from preeclamptic pregnancies were found. D) Representative image of VEGF present in the conditioned medium (CM) of hCMEC/D3 exposed (24 h, 1%, v/v) to umbilical cord plasma from preeclamptic pregnancies. Ponceau staining was used as a loading control. E) No significant differences were found in VEGF levels in the CM between experimental groups (T-test, n=9 per group, p=0.24). Each dot represents one study subject. Values are presented in the median ± interquartile range. *p<0.05.
